# Assessing the Effectiveness of Climate-Smart Health Facilities in Small Island Caribbean Nations

**DOI:** 10.5334/aogh.4755

**Published:** 2025-08-19

**Authors:** Maureen Lichtveld, James Hospedales, Spencer Reed Davenport, Jeanine Buchanich, Judith Harvey, Firoz Abdoel Wahid, Loren De Freitas

**Affiliations:** 1 University of Pittsburgh School of Public Health, Pennsylvania, USA; 2 EarthMedic/Nurse Foundation for Planetary Health, Trinidad and Tobago; 3 International PAHO Consultant (IPC) on the Smart Hospitals Initiative, St Lucia; 4 Department of Public Health and Primary Care, the University of the West Indies, Trinidad and Tobago

**Keywords:** climate adaptation, smart health facilities, Caribbean region

## Abstract

*Background:* The small island developing states (SIDS) in the Caribbean are particularly vulnerable to the impacts of climate change. Many SIDS’ health facilities are in high-risk areas such as coastal zones and are affected by extreme weather events. It is imperative to develop climate-resilient health systems to ensure health service continuity during and after an extreme weather event. One model to achieve this is the Smart Hospital Initiative.

*Objective:* This case study was designed to strengthen the evidence base for decision-making regarding investing in Smart Hospital Initiative facilities as a climate adaptation strategy.

*Methods:* This case study used secondary data derived from the Smart Hospital Initiative implementation (*n* = 55) focusing on four domains: country population/population served by the facility; pre-post Smart Hospital Initiative facilities' data; disaster and severe weather events’ data; and diabetes mellitus (DM) mortality data. To assess the effectiveness of the initiative, an analysis of these data domains across seven countries is presented.

*Findings:* Examining population size and healthcare service resources, healthcare facilities’ readiness, climate-related disasters, and a health condition of concern, represents a viable strategy to assess the impact of climate adaptation on health. The Hospital Safety Index data showed that there were statistically significant pre-post retrofitted smart improvements across all 55 retrofitted facilities. The findings revealed that the effectiveness of any adaptation strategy is influenced by local financial and human resources beyond an initial, often external, investment and the capability to maintain the initial retrofitting of health facilities’ impact on DM mortality.

*Conclusions:* Climate-smart hospitals are a promising initiative to support the development of climate-resilient health facilities in SIDS. However, successful implementation depends on local capacity to support implementation and maintenance. We propose a framework to assess the utility of implementing climate-smart facilities as an adaptation strategy.

## Introduction

The small islands and low-lying states of the Caribbean comprise some 30 countries and territories with 40 million English-, Spanish-, French- and Dutch-speaking people. All are extremely vulnerable to the adverse impacts of climate change by virtue of their location in the hurricane belt, small size, limited resources, and stretched healthcare systems. During 2000–2019, nine of the top ten countries or territories with losses from disasters were in the Caribbean (UNDRR 2020) [1–5]. Given the geography of the Caribbean, most health facilities in the region are in coastal areas or on slopes and thus at risk from electrical outages, storm surge, and landslides during hurricanes and tropical storms. The overall *goal* of this case study is to examine the impact of climate change adaptation strategies of health facilities on the continuity of health services in selected Caribbean small islands.

### Smart hospital initiative description

In response to previous disasters, the Pan American Health Organization (PAHO) launched the Smart Hospitals Initiative Phase 1 in 2009 and completed it by 2014. The adaptation strategy described in this case study is the Smart Hospitals Initiative Phase 2. Smart hospitals are resilient facilities able to function within a health network or independently of the network to a large extent after disasters. Once retrofitted as smart, these facilities are defined as being safe against expected natural hazards, environmentally friendly in their operations, and well maintained. These facilities include hospitals, health centers, and units such as central storehouses for medical supplies, which contribute to the access to and quality of care.

The Smart Hospitals Initiative components include training of stakeholders, assessments, surveys related to the project goals, selection of health facilities for retrofitting, design, and construction of renovations to these existing health facilities, monitoring the project’s impact and maintenance-related interventions immediately after retrofitting. A summary of the core components of the initiative is presented in Table 1.

**Table 1:** Summary of the core components of the Smart Hospital Initiative.

PROJECT PHASE	COMPONENT	SPECIFIC ACTIVITIES	TIMELINE
1	Assessments	HSI status for safety, development of Green Checklist to establish the level of greening	2009/2014 Pre- and post- retrofitting
1	Retrofitting	Design and construction of improvements to infrastructure	2009–2014
2	Assessments	HSI and Green Checklist for status in terms of safety and greenness	2015–2017 depending on country
2	Site selection	Cost-effectiveness analysis study of proposed retrofits based on the contribution of safety and greening works	2016–2017 depending on country
2	Training	Preparatory training for constructors and for staff to enhance familiarity with new systems; emergency planning skills	2016–2018
2	Retrofitting	Design, tendering and construction	2017–2023, interrupted by Hurricane Maria and COVID-19
2	Training	Focus on maintenance and care of installations	2019–2022
2	Surveys and evaluation	Impact of the project	2020–2023
2	Outreach	Sharing of best practices	2022 onwards

Phase 1 of the Smart Hospitals Initiative was a pilot project conducted in St. Kitts and Nevis and St. Vincent and the Grenadines [6, 7].

Phase 2 project implementation took place between 2015 and 2023, with training of 1,266 persons, and assessments of over 415 facilities in seven countries (Belize, Dominica, Grenada, Guyana, Jamaica, St. Lucia, and St. Vincent and the Grenadines) preceding the first contract awards in 2018. Construction continued through the COVID pandemic until 2023. Over 55 facilities were retrofitted with funding from the UK Foreign Commonwealth and Development Office. As human-caused climate change advances, hurricanes intensify more rapidly, are stronger, wetter, and linger longer. Combined with other factors like rising sea levels, hurricanes are causing more damage, destruction, injury and death, making the Smart Hospital Initiative even more needed [8–10].

A pre-existing tool, the Hospital Safety Index (HSI), was used to evaluate what was needed to make the facilities safe against natural hazard events [11]. “Green” elements, such as rainwater harvesting and renewable energy, were integrated by means of a new tool, the Green Checklist, thus reducing greenhouse gases, while increasing resilience to electricity and water supply failures. The Smart Hospital Initiative “gold standard” of A/70 was defined as an “A” rating on the HSI scale and 70% on the green scale.

Following assessments using the HSI and the Green Checklist, facilities were chosen for upgrade to the A70 Standard or for targeted small-scale interventions. Inpatient facilities were generally chosen for upgrade to A70, while primary care facilities at strategic points in the health network received targeted interventions. The latter facilities would not be expected to be open during a predictable hazard event and may suffer minor damage needing repair after the event. They would serve as focal points for providing care to affected populations immediately after a disaster.

Implementation was a collaboration between PAHO and the Ministries of Health in the target countries. The staff were engaged before retrofitting in discussions relating to the proposed scope of work. During retrofitting, they participated in regularly scheduled site meetings, and after retrofitting, the feedback they gave informed the activities in the Defects Liability period, referring to the time after construction is substantially complete, typically 6 months to 1 year, in which the contractor must remedy any flaws in workmanship or materials that are discovered.

The Smart Hospitals Initiative was in keeping with local policy priorities to the extent that countries have continued to scale up the program using funding from other donors (e.g., the EU in Belize) and using loans (e.g., World Bank loans to OECS countries). The experience gained in Phase 2 was used to justify the applications for funding to scale up the initiative. The research described herein contributes to the literature on the subject and can be used in future planning.

### Adverse health outcome of concern

Caribbean populations are aging and have a high prevalence of noncommunicable diseases (NCDs), including diabetes, asthma, heart disease, and cancer. Improvements in NCD outcomes have been slower in the Latin American and Caribbean (LAC) than in the Organization for Economic Co-operation and Development (OECD) countries [12]. Blood glucose levels that are higher than optimal, even if below the diagnostic threshold for diabetes, may, over time, impact organ system functioning, and, left uncontrolled, may advance to diabetes mellitus (DM) morbidity [13]. The risk of macro-vascular disease increases well before the diagnostic criterion for diabetes of fasting plasma glucose ≥ 7.0 mmol/L, which is selected on the basis of micro-vascular complications such as diabetic retinopathy. Between 2010 and 2019, deaths attributable to high blood glucose increased by 8% in LAC while they decreased by 14% in the OECD [12]. During this time period, the average prevalence of adult diabetes in LAC countries increased from 7.4% to 9.7% [12]. The prevalence of both diabetes and mortality attributable to high blood glucose is higher than the LAC average in Antigua and Barbuda, Barbados, Belize, Brazil, Guyana, Jamaica, St. Lucia, St. Vincent and the Grenadines, Suriname, and Trinidad and Tobago [12]. Among the Caribbean countries (33 countries), 12 had higher prevalence rates than the regional average, with the highest in Belize at 17% [12]. In 2019, the country with the highest mortality was Guyana (188 deaths per 100 000 population), followed by Suriname and St. Vincent and the Grenadines (155 and 153, respectively) [12].

This climate adaptation case study was designed to strengthen the evidence base for decision-making regarding investing in smart facilities as an effective climate adaptation strategy. Based on the findings of this case study, we propose a framework to assess the utility of implementing climate-smart facilities as an adaptation strategy in other geographic areas and in the context of other climate-related impacts on health and the environment.

## Methodological Approach

### Study design and setting

The study design was based on available secondary data for four data domains: the country population and the population served, pre-post Smart Hospital Initiative facilities’ data; disaster and severe weather events’ data, and DM health data. Table 2 provides a summary of the number of facilities, the country populations, and populations served by the Smart Hospital Initiative.

**Table 2:** Summary of population data for the selected countries.

COUNTRY	NUMBER OF FACILITIES THAT BENEFITTED	CURRENT COUNTRY POPULATION (2022)	CATCHMENT POPULATION FOR SMART HOSPITAL PROJECT
Belize	6	405,273	88,600
Dominica	7	72,734	27,355
Grenada	5	125,433	105,897
St. Vincent and Grenadines	8	103,955	39,010
Guyana	5	808,728	117,798
Jamaica	14	2.8 million	307,729
St. Lucia	16	179,861	57,763

The Smart Hospitals Initiative collected data from 2015 to 2023 across seven countries in the Caribbean. HSI comprises three elements: structural (e.g. foundation, walls, roof strength), non-structural (e.g., water, electricity, telephone), and functional (e.g., staff training, medication access, working disaster plan).

Data for the Smart Hospital Initiative were collected as structural, non-structural and functional aspects of the facility and its operations. For this case study, 21 questions were selected that were considered pertinent to the continuity of healthcare after emergencies (Table 3). The full questionnaire of 96 questions can be found in Appendix A.

**Table 3:** Summary of the Smart Hospital HSI data.

HSI CONSTRUCT	HSI AREA OF ASSESSMENT	QUESTION NUMBERS	DETAILS COVERED	DETAILS NOT INCLUDED
Non-structural	Alternative sources of electricity	15, 16	Condition, safety, capacity available	Maintenance, restoration after emergency
Non-structural	Alternative source of water and water reserves	24, 26	Condition, capacity available. Storage of mains supply and other non-potable sources	Location/safety of placement, maintenance, restoration after emergency
Non-structural	Solid waste disposal systems	35	Hazardous and non-hazardous waste systems’ capacity, compliance with regulations and maintenance	
Non-structural	Lab and medical equipment	45	Condition, capacity, and range of equipment that are operational	Maintenance and restoration after an emergency
Non-structural	Fire protection system	56	Reinstatement after emergency	Details of the fire safety equipment
Non-structural	Access and evacuation routes	57, 58, 59, 60	Access and exit routes free of obstacles and signposted	
Functional	Documented disaster plans; emergency drills	67, 69, 86, 87	Specified actions to be taken, including provision for high-level hazards	
Functional	Emergency protocols	74	Admittance and treatment of patients	
Functional	Communication with the media and the public	80	Responsibilities	
Functional	Supplies for use during an emergency	88, 89, 90	Medication, instruments, sterilized equipment, life support equipment	Basic supplies for staff, e.g. food, toilet articles
Functional	Intra-hospital epidemiologic surveillance	92		

Electronic copies of the data were obtained with permission from the PAHO Regional office in Barbados. For the implementation of the Smart Hospital Initiative, there were written agreements between the respective countries’ Ministries of Health and the PAHO. Hard copies of the data were stored in the main office of the implementing agency, PAHO. Since structural changes were not made to all facilities, only non-structural changes that include all facilities are included in Table 3.

### Data collection

Data collection involved the extraction of data in four areas: disaster and severe weather events, diabetes mortality data, Smart Hospital Initiative data, and population served by the health facility.

### Data sources

#### Disaster and severe weather events data

Data on major disasters and weather events from 2014 to 2024 were extracted for each of the seven Caribbean countries studied. These events consisted of hurricanes, tropical storms, floods, landslides, wildfires, and volcanic activity. The list of events was collected from the National Oceanic and Atmospheric Administration Historical Hurricane Tracks and Office for Coordination of Humanitarian Affairs ReliefWeb databases and the United Nations Office for Disaster Risk Reduction 2000–2022 disaster report for the region [14, 15]. Additional damage and impact statistics were collected from the International Federation of Red Cross and Red Crescent Societies Disaster Response Emergency Fund reports found through ReliefWeb for each event and from local government reports and news. Impactful events (i.e., considering the number of individuals affected, power outages, and both total damage and damage to healthcare facilities) were identified for inclusion in the overall analysis.

#### Diabetes mellitus mortality data

DM death rates were extracted from country-specific public sources based on the data available [16, 17]. Different metrics were used for different countries based on how the data were reported for each country. These are outlined as follows:

- Belize, Guyana, Jamaica: Annual age standardized DM death rates (per 100,000 standard population) for Belize, Guyana, and Jamaica from 2000 to 2019 were obtained from PAHO [16].
- Grenada, St Lucia, St. Vincent, and the Grenadines: Age-adjusted DM death rates (per 100,000 standard population) from 2000 to 2021 were obtained for Grenada, St. Lucia, and St. Vincent and the Grenadines from WHO [17].
- Dominica: DM deaths as a percentage of total deaths were obtained from the WHO from 2000 to 2020 [17].

DM death rates for each country were graphed and examined congruent with a timeline of natural disasters and retrofitting to observe potential changes in trends [2, 14, 15].

#### Hospital safety index data

A summary of the HSI scoring system used is presented in Table 4. Individual HSI questions were answered as a categorical rating: “Low,” “Average,” or “High” and encoded as 1, 2, 3 for analyses. The HSI forms were completed by trained assessors who visited the facilities, made observations, and interviewed the facility staff. Changes in the HSI category for each facility were analyzed pre- and post-retrofitting to assess the impact of adaptation on a facility’s readiness for a natural disaster. The country-specific value changes were averaged for all 55 facilities included in the analysis (Table 2). Six facilities that were designed by the project teams but were not funded for construction were excluded from the analyses.

**Table 4:** Summary of HSI Scoring System.

SAFETY INDEX	CATEGORY	EVALUATION
1	Low	Unlikely to function
2	Average	Likely to function
3	High	Highly likely to function

### Data analysis

#### Analysis of weather and DM mortality data

**Figure 1:**
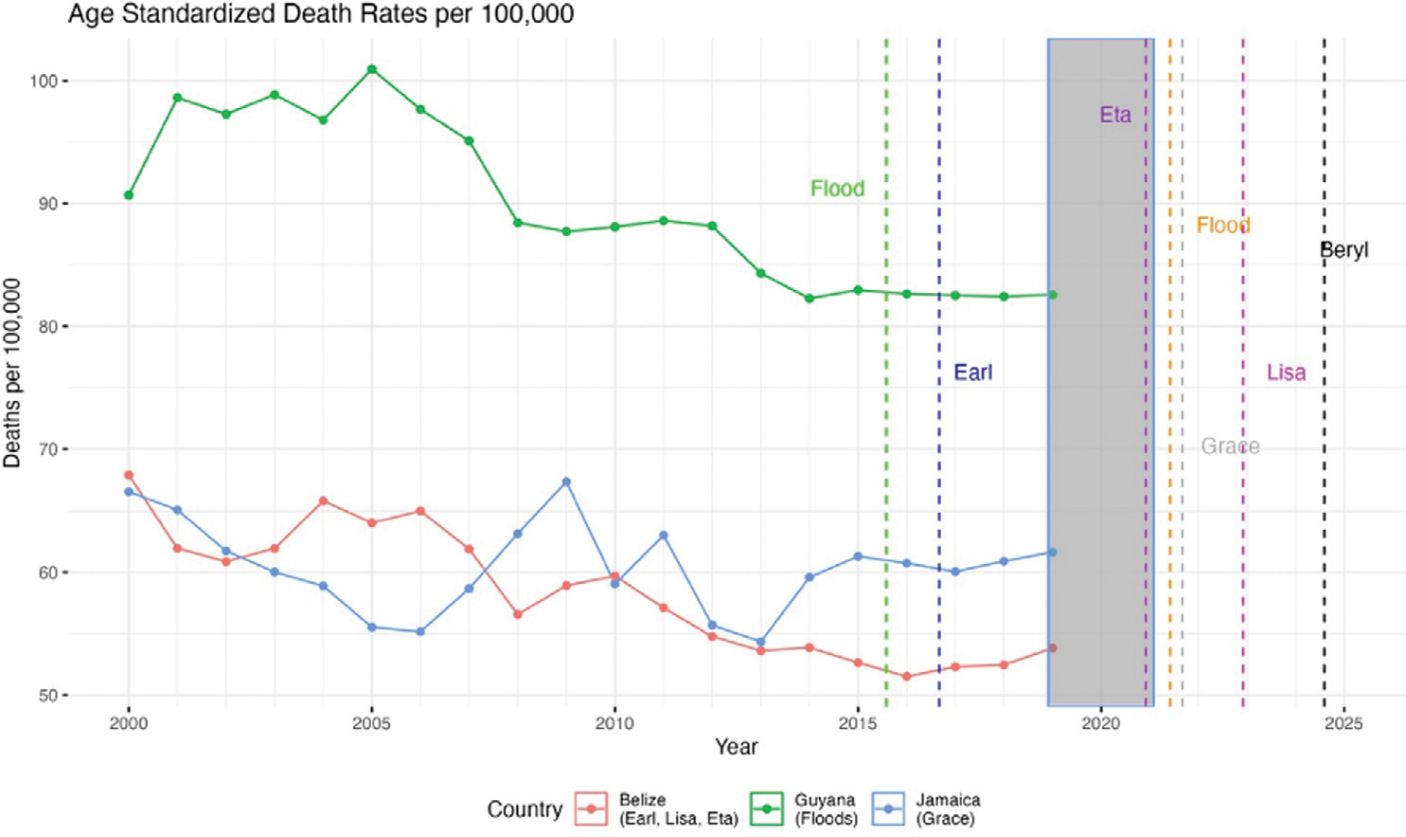
Age standardized DM death rates per 100,000 for Belize, Gand Jamaica (PAHO). Natural disasters are parenthesized adjacent to the country impacted.

**Figure 2:**
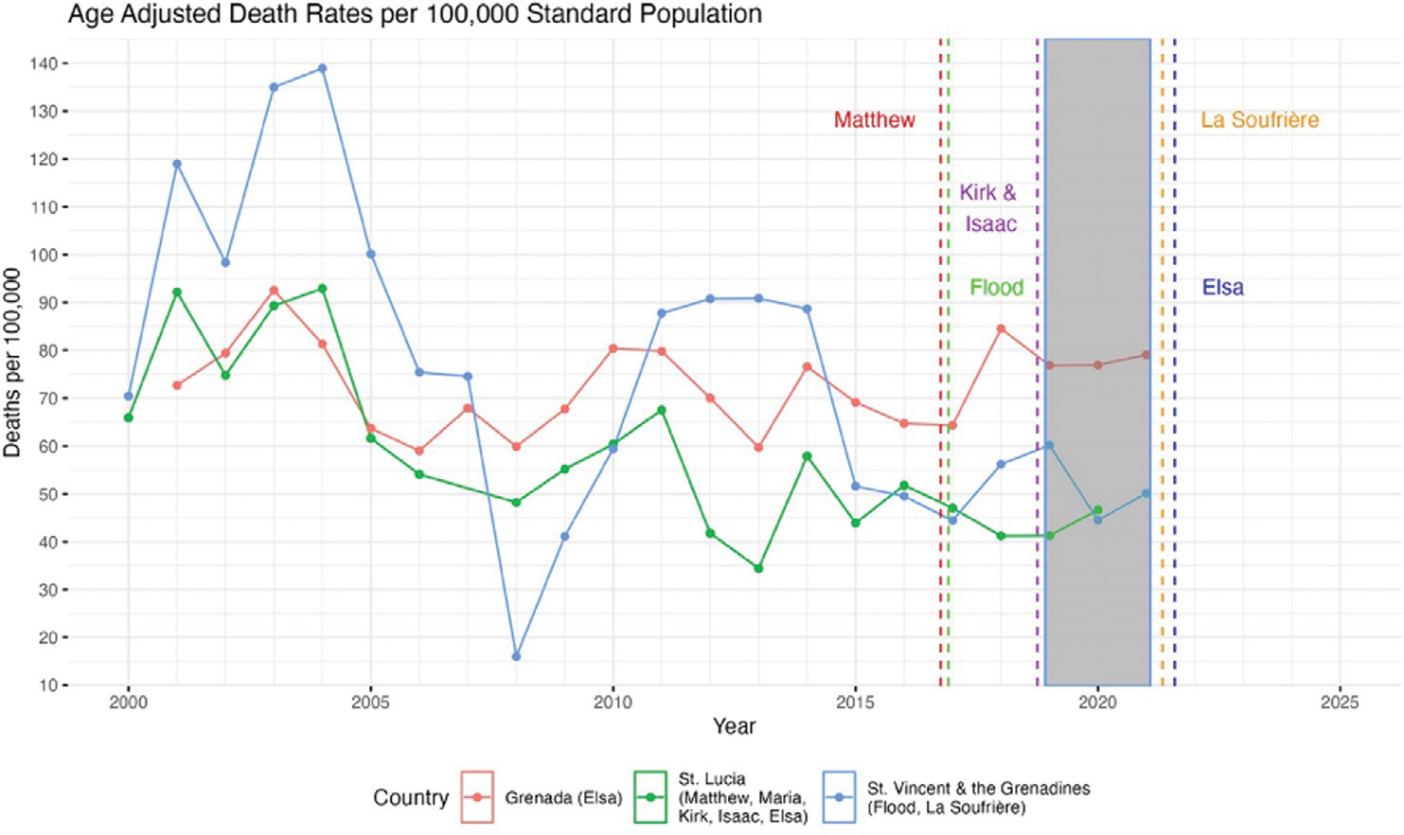
Age adjusted DM death rates per 100,000 standard population for Grenada, St. Lucia and St. Vincent and the Grenadines (WHO). Natural disasters are parenthesized adjacent to the country impacted.

**Figure 3:**
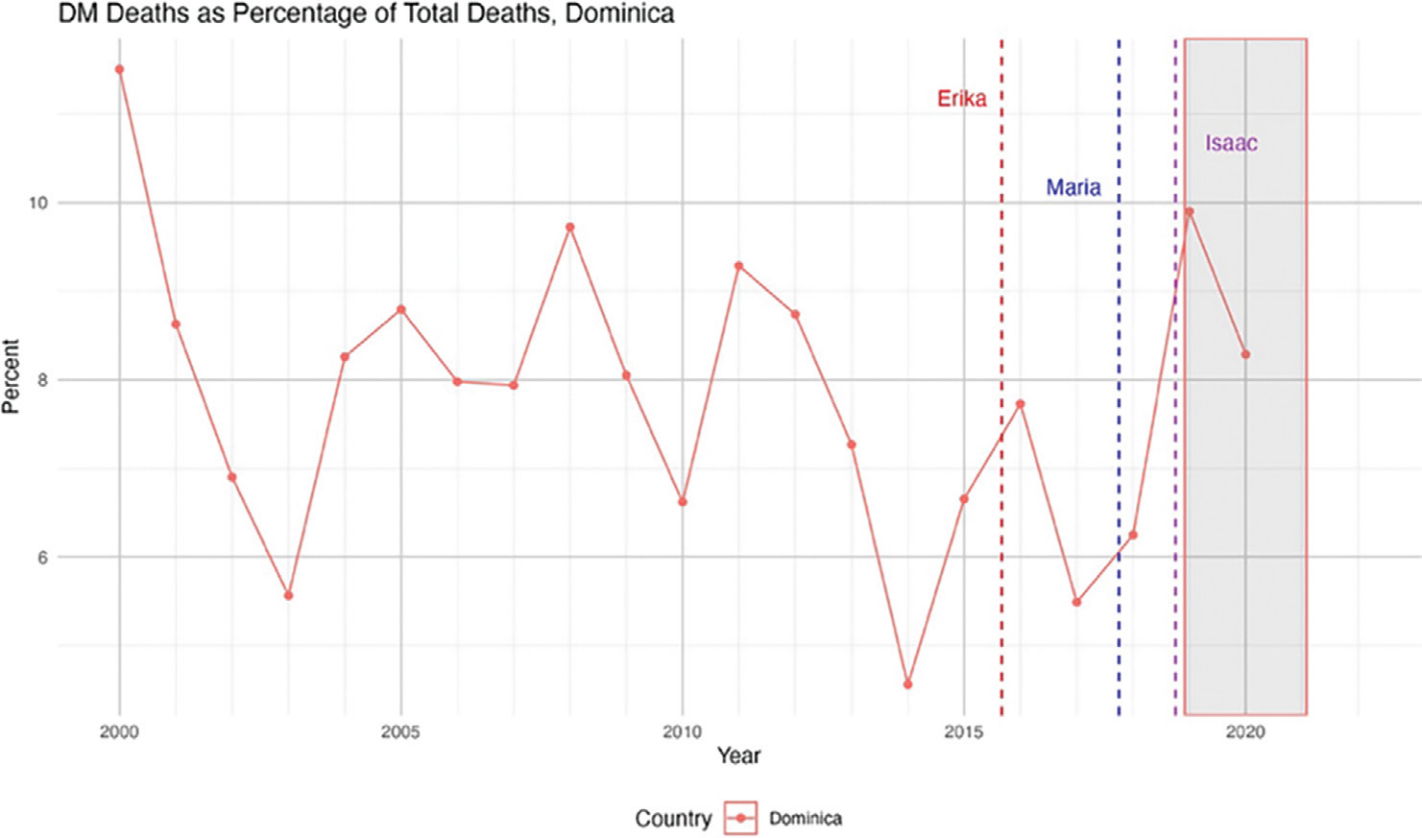
DM deaths as a percentage of total deaths in Dominica. Natural disasters are parenthesized adjacent to the country impacted.

Graphical analyses were conducted to assess the relationship between severe weather events and the change in diabetes mortality before, during, and after retrofitting (Figures 1–3).

#### Analysis of hospital safety index data

**Figure 4:**
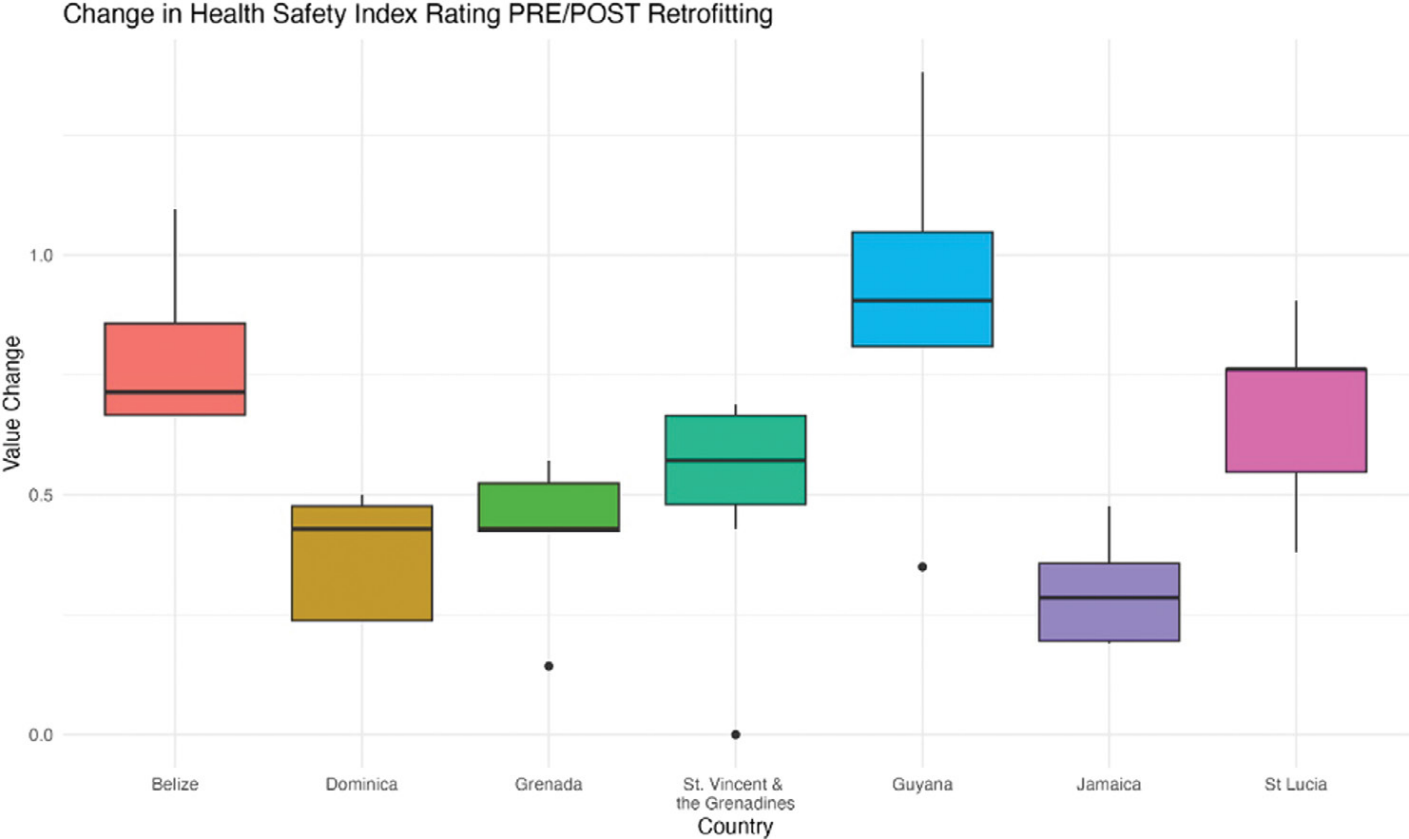
Country-specific categorical change in HSI ratings before and after Smart retrofitting. Each line indicates a question from the HSI form for a facility and its corresponding rating.

To determine if the change in HSI scores differed by country, the HSI scores were recorded for every facility before and after retrofitting. The changes in HSI scores were then found by taking the difference between the corresponding before and after scores. The change in the HSI scores for all facilities in each country was then averaged and plotted in Figure 4. An analysis of variance test (ANOVA) was used to identify if there was a statistically significant difference in mean change between countries.

**Figure 5:**
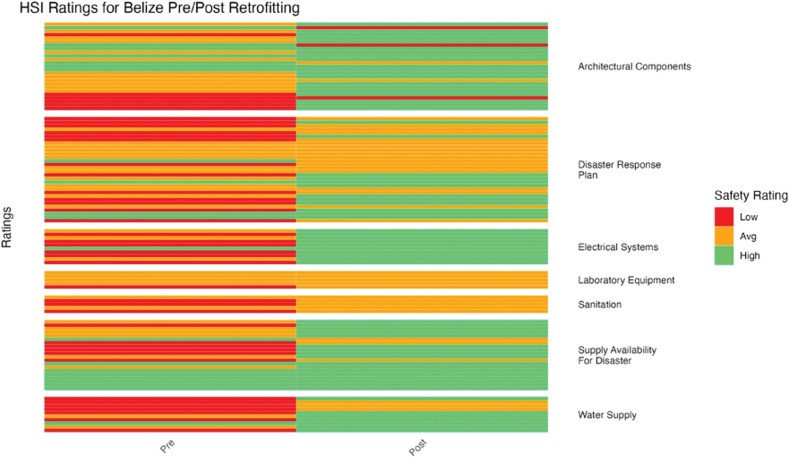
Belize HSI before and after retrofitting changes in facility ratings (5 facilities represented).

**Figure 6:**
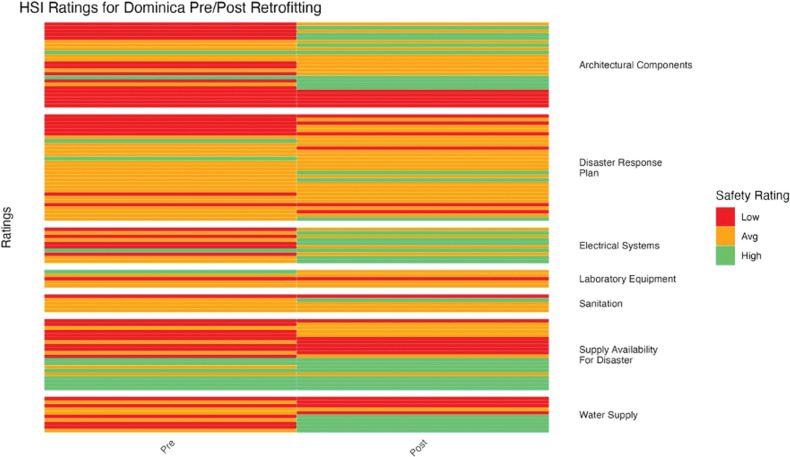
Dominica HSI before and after retrofitting changes in facility ratings (5 facilities represented).

**Figure 7:**
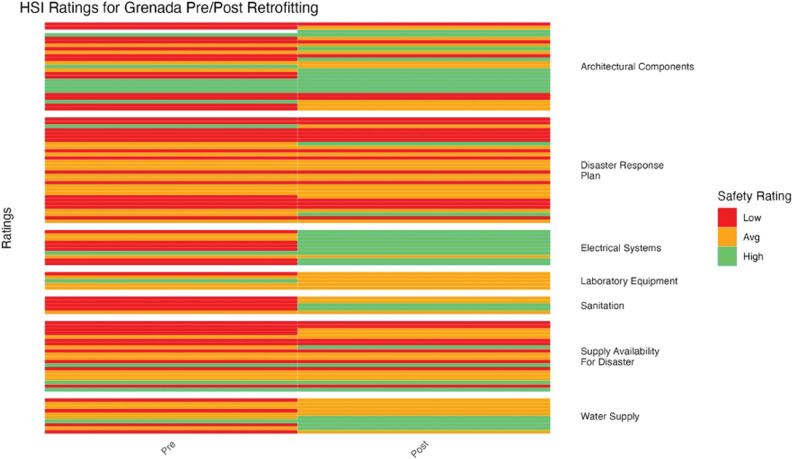
Grenada HSI before and after retrofitting changes in facility ratings (5 facilities represented).

**Figure 8:**
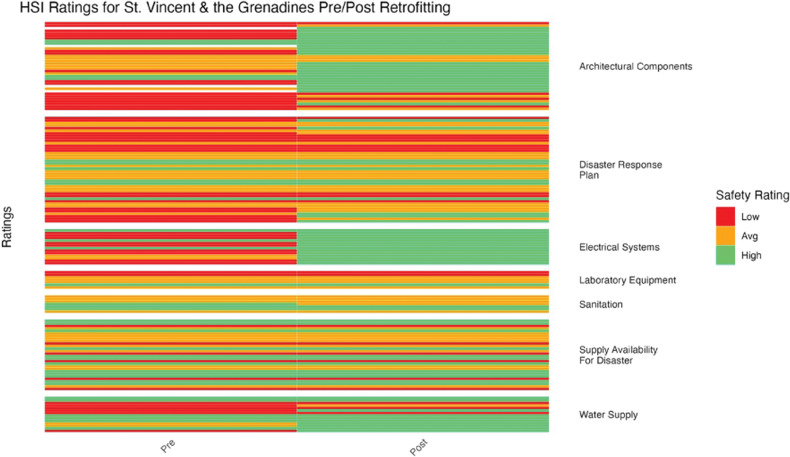
St. Vincent and the Grenadines HSI before and after retrofitting changes in facility ratings (7 facilities represented).

**Figure 9:**
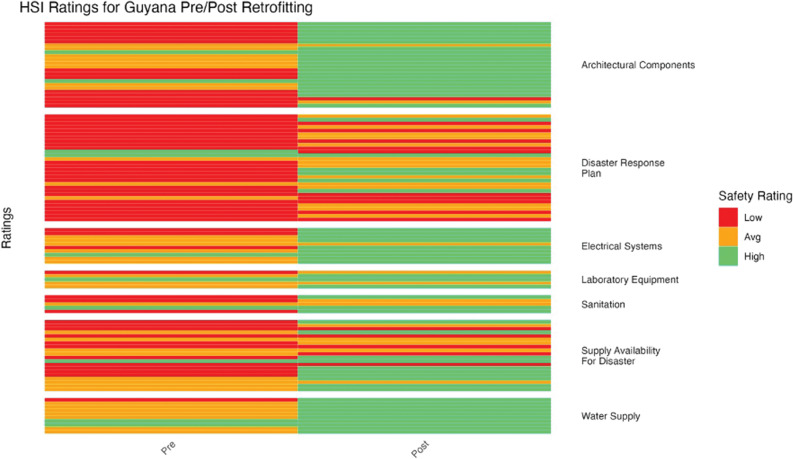
Guyana HSI before and after retrofitting changes in facility ratings (5 facilities represented).

**Figure 10:**
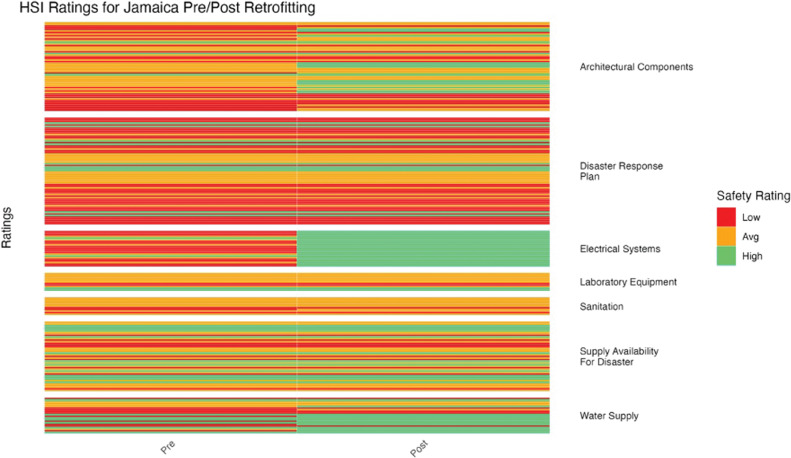
Jamaica HSI before and after retrofitting changes in facility ratings (12 facilities represented).

**Figure 11:**
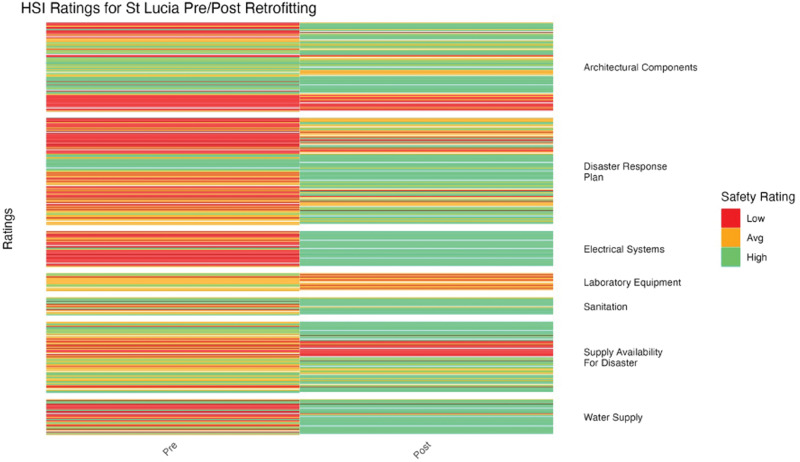
St. Lucia HSI before and after retrofitting changes in facility ratings (15 facilities represented).

To determine if there was a statistically significant difference in mean HSI ratings before and after retrofitting, a paired *t*-test was conducted (Figures 5–11). Data analyses were conducted using R Studio v.2023.12; data management was handled in PyCharm v2023.3.3. Statistical significance was assessed as *p* < 0.05.

## Findings

### Integration of weather and diabetes data

Figures 1–3 show diabetes mortality data for the seven Caribbean countries selected by juxtaposing major hurricanes and other severe weather events. The gray bar in Figures 1–3 show the time period when the retrofitting of inpatient care hospitals to smart facilities was accomplished.

DM death rates for Belize and Guyana decreased over the course of the time period examined, with a leveling off in Belize since 2015. Jamaica had an initial decrease in DM death rates but increased in 2013 with rates since then similar to those of 2010. DM data were not available to assess the impact of disasters post-2019 (Figure 1).

DM death rates were relatively stable for Grenada and slightly decreased for St. Lucia. Rates for St. Vincent and the Grenadines were more unstable with an anomalously low rate in 2008 (Figure 2). DM data were available until 2022.

DM deaths as a percentage of total deaths have variability and are generally unstable (Figure 3).

### Hospital safety index values pre- and post-retrofitting

Many countries showed similar improvements in HSI rating categories before and after retrofitting of between 0.6 and 0.8 (less than 1 unit change across all facilities included per country). Guyana had the most improvement, while Jamaica had the lowest improvement (0.3) (ANOVA *p*-value = 9.26 × 10^–7^) (Figure 4).

Across all countries combined, there was a statistically significant improvement in HSI ratings before and after retrofitting (paired *t*-test *p*-value = 2.2 × 10^–16^) (Figures 5–11). HSI ratings improved significantly after retrofitting for Belize (*p* = 6.08 × 10^–4^). Electrical systems improved from a majority of low ratings to entirely high ratings. Laboratory equipment and sanitation showed the least improvement (Figure 5).

HSI ratings varied in improvement for Dominica (Figure 6). Architectural components, electrical systems, and water supply showed the most improvement, while laboratory equipment, sanitation, and the disaster response plan showed the least improvement (*p* = 2.83 × 10^–3^).

HSI ratings for Grenada improved moderately (*p* = 4.92 × 10^–3^) (Figure 7). Architectural components, electrical systems, and water supply showed the most improvement, while the disaster response plan and supply availability remained relatively similar.

HSI ratings demonstrated varying improvement for St. Vincent and the Grenadines after retrofitting (Figure 8). Architectural components, electrical systems, and water supply showed the most improvement. Electrical systems improved from almost entirely low ratings to all high ratings. The disaster response plan and supply availability showed little or no change (*p* = 1.45 × 10^–3^).

HSI ratings improved significantly overall after retrofitting for Guyana (*p* = 5.88 × 10^–3^) (Figure 9). Architectural components, electrical systems, laboratory equipment, sanitation, and water supply all improved from a majority of low and average ratings to a majority of high ratings.

HSI ratings improved variably after retrofitting for Jamaica. Electrical systems saw the greatest improvement, moving from a majority of low ratings to entirely high ratings (Figure 10). Architectural components and water supply showed moderate improvement, while other categories remained relatively unchanged (*p* = 2.53 × 10^–6^).

HSI ratings improved significantly after retrofitting for St. Lucia (*p* = 7.28 × 10^–10^) (Figure 11). Laboratory equipment did not show much improvement, while all other categories improved considerably. Electrical systems had the clearest improvement, from a majority of low ratings to entirely high ratings.

## Discussion

### Addressing key health risks through the smart hospitals initiative

The Smart Hospitals Initiative holds great potential as an important adaptation strategy in the Caribbean, addressing prevalent NCD risks in the face of climate-related disasters. The diabetes mortality data plotted against the timeline of the initiative suggest that there was continuity of care during and after extreme weather events. This is essential for managing acute as well as chronic conditions such as DM, the “indicator” disease chosen for this case study.

While the extent of improvement across countries was varied, the data showed that retrofitted facilities improved in structural and functional safety and the safety of non-structural elements. This improved preparedness enhanced the overall functionality of health facilities, which in turn potentially reduced disruptions in healthcare services. Reductions in diabetes-related mortality in countries such as Belize and Guyana may show promise of a potential association with providing care in smart facilities. This is suggestive that resilient healthcare infrastructure can contribute to better health outcomes for populations highly burdened with NCDs. However, the available secondary data are limited to confirm this relationship.

### Social, economic, behavioral, and institutional drivers

The success of the Smart Hospitals Initiative may also be related to its multi-level and multi-sectoral approach. The engagement of local communities and healthcare staff was essential, both in the planning and implementation phases. This collaboration ensured that local needs and challenges were incorporated into the adaptation process, which increased the relevance and effectiveness of the interventions.

### Contributions to scientific understanding and future research opportunities

This case study contributes significantly to the understanding of climate change adaptation in healthcare, particularly in the context of small island developing states (SIDS). It highlights the importance of integrating health facility resilience into climate adaptation strategies. The findings may indicate that resilient healthcare infrastructure, such as smart hospitals, is better adapted to the impacts of climate-related disasters. However, the variability in secondary disease-related data across the countries studied emphasizes the need for further research. For example, no secondary DM morbidity data were available.

DM morbidity data along with other factors that influence health outcomes (e.g., broader socio-economic and healthcare system factors) beyond just infrastructure improvements would provide a more advanced opportunity to assess the role of smart facilities in preserving continuity of care post climate-related events.

The growing burden of NCDs in the Caribbean, driven by aging populations and social inequalities, calls for a more comprehensive approach that combines strengthening healthcare systems with public health efforts aimed at disease prevention. Future research should explore the long-term effectiveness of climate-smart facilities, using morbidity data alongside mortality statistics to assess continuity of health services.

### Relevance to other countries and contexts

The Smart Hospitals Initiative illustrates the potential for infrastructure adaptation to mitigate climate-related health risks and is relevant to other SIDS and LMICs facing similar NCD burdens and climate-related challenges. However, the success of such interventions is dependent on local capacity for implementation and sustainability. In countries with weaker healthcare systems or lower financial resources, modifications to the scope of retrofitting may be necessary.

### Proposed framework to assess the utility of smart health facilities

While improvements in facility resilience were evident, the direct relationship between these improvements and reductions in adverse health outcomes (in this case, limited to available data on DM mortality) remains complex and difficult to establish definitively, due to data limitations. However, the findings of this case study are instrumental in developing a framework to assess the utility of implementing climate-smart facilities as an adaptation strategy. Here we present a potential framework that may be used to assess the utility of smart health facilities as part of future assessments.

**Figure 12:**
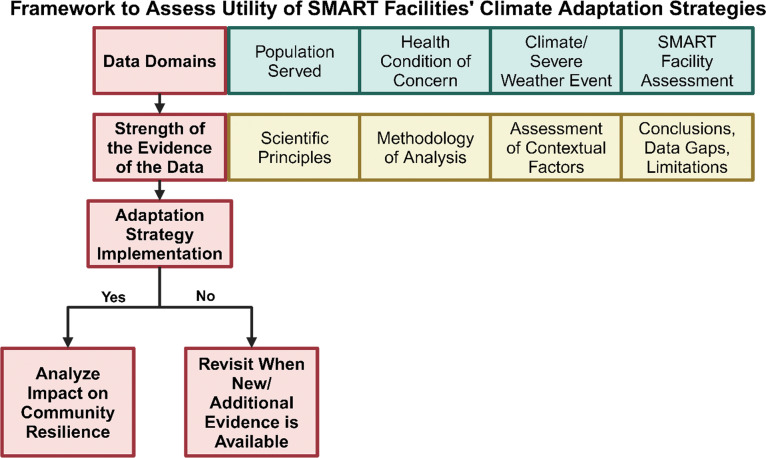
Framework to Assess Utility of Smart Facilities’ Climate Adaptation Strategies (Created with BioRender.com).

Figure 12 outlines a three-tiered framework to assess the utility of implementing climate-smart facilities as an adaptation strategy for future risk benefits assessments of climate adaptation efforts. In Tier 1, we identify the four data domains we selected to assess the impact of Smart Hospital facilities as an adaptation strategy as described in previous sections of the case study. The second tier represents an assessment of the strength of the evidence for each of these data domains focused on four areas. Based on the findings of the assessment conducted in Tier 2, a decision can be made whether to proceed with implementing smart facilities as a useful climate adaptation strategy. If the decision is to proceed, then the next step is to determine if the benefit and contribution to community resilience are worthy of the investment. This proposed framework is developed based on data from seven countries in the Caribbean region. By expanding this tiered analysis strategy beyond the Caribbean, the framework could help guide future climate adaptation policies in healthcare systems globally, particularly in other low- and LMICs facing rising climate risks.

In this instance, the framework can be a useful tool to assess the utility of an alternate climate adaptation strategy, including constructing a new inpatient facility according to evidence-based smart technology, instead of retrofitting an existing one.

### Strengths of innovations of the current study

This is the first study of its kind that attempts to assess the effectiveness of climate-smart health facility intervention to improve public health outcomes in highly vulnerable SIDS. The findings are promising, but further research is needed which looks at climate events and impacts on smart health facilities in relation to health outcomes, such as DM mortality or all-cause mortality.

### Limitations

The Smart Hospital Initiative intervention was designed to improve the safety and resilience of health facilities, and the pre- and post-data interventions are promising in achieving those outcomes. The project was not designed to show improvement in public health outcomes, though it might be hypothesized that improved safety and resilience should lead to improved continuity of access to care. The lack of a standardized selection methodology hampers facility to facility comparisons. Also, smart hospitals focus on improving infrastructural resilience. Especially in LMICs, ongoing maintenance and the sustainability of these upgrades can be a challenge.

### Data availability on public health outcomes such as diabetes mortality

The Smart Hospital Initiative covered less than half the population in the intervention countries, except Grenada, where over 80% of the population was covered. This makes the interpretation of national mortality data trends very challenging. What would be needed is local-level data, e.g., all-cause mortality and NCD-specific mortality, in relation to climate events.

Concurrent trends in risk factors in populations are also potential confounders, e.g., increased obesity that drives up diabetes incidence and prevalence.

- The approach used by the team was to show that the HSI scores before and after can predict the resilience of health facilities.
  1. The use of certain questions means that the overall HSI assessment score was not used as a measure of resilience, as PAHO intended. Questions are given different weighting depending on their importance, but all questions are components of resilience in PAHO's framework. The research team selected questions to make the analysis more focused on certain critical issues and more relevant to the case of facilities with limited interventions.
  2. The amount of retrofitting varied depending on the facility. Some were gold standard retrofits, and some were limited interventions. The study lumped these together in the analysis and categorized the outcome by country. This means that countries with only gold standard retrofits will appear more resilient (Belize) than countries with many limited intervention retrofits (Jamaica). It does not account for the network approach.
- The study proposal was to show that deaths increased in hurricane years, and this could have been caused by a lack of continuity of healthcare, or vice versa, that hurricane deaths were not as high as they could be, due to better continuity in healthcare provision.
  Country data are limited, in terms of cause of death, location of the people affected, years of the data sets. Most countries did not have electronic records of patient data. The correlation between deaths and continuity of healthcare provision is not as strong as the case study was intended to demonstrate due to data limitations.
  1. In PAHO records, the catchment population is quoted by the facility, because the whole country does not benefit from improved continuity of healthcare. However, only country-wide data for deaths and health outcomes were available to the research team. The location of people is important to strengthen this correlation.
  2. Only the early years of the Smart Hospital Initiative are covered by the data presented for deaths and health outcomes. Many of the retrofits were not in operation during the time period of available DM mortality data. Re-opening dates could be a better guide to the “end of retrofit,” and these would be in the records of individual Ministries of Health. These data have not yet been collected in a standardized fashion and reported to PAHO.
  3. DM mortality was the chosen indicator. Data were not available to assess the role of resilient facilities in ensuring the continuity of DM health services, e.g., through the continued provision of insulin, access to renal dialysis, and other diabetes-related services.
- Recommendation of workforce development as a critical component of the Smart Hospital Initiative
  1. Healthcare providers in the Caribbean and many other LMICs and high-income countries alike lack the knowledge and skills beyond their clinical training to provide continuity of care and services during and in the immediate aftermath of climate-related disasters and severe weather events. Knowledge and skills in climate-related adverse health conditions are a critical component of assuring continuity of care and health services.
  2. In addition to clinical skills, it is recommended that public health research skills be a prerequisite to conducting epidemiologic trend analyses of the impact of resilient facilities on health outcomes over time.

## Conclusion

This case study evaluated the implementation and effectiveness of the Smart Hospital Initiative to support the development of climate-resilient health systems in seven Caribbean islands. Research to comprehensively assess the role of smart facilities in assuring continuity of care requires a four-pronged strategy: (1) systematic collection and analysis of the four data domains—population, weather, healthcare facilities, and health outcome data; (2) regional disease surveillance systems built on standardized and well-resourced country-level data; (3) a regionally standardized methodology for selecting and retrofitting health facilities using a community-centered approach; and (4) a skilled climate and health workforce. A transdisciplinary data science approach will promote research translation into frontline practice, targeting the most vulnerable, and can accelerate achieving climate-resilient communities in the Caribbean region.
